# Supplementary material for: A tumor-targeted trimeric 4-1BB-agonistic antibody induces potent anti-tumor immunity without systemic toxicity
Source: Nat Commun. 2018 Nov 15;9:4809. doi: 10.1038/s41467-018-07195-w (PMC6237851; doi:10.1038/s41467-018-07195-w)
Supplement: Supplementary file 1 — Supplementary Information [file 41467_2018_7195_MOESM1_ESM.pdf]

Supplementary Information

A tumor-targeted trimeric 4-1BB-agonistic antibody induces potent anti-tumor immunity without systemic toxicity

Compte *et al*.

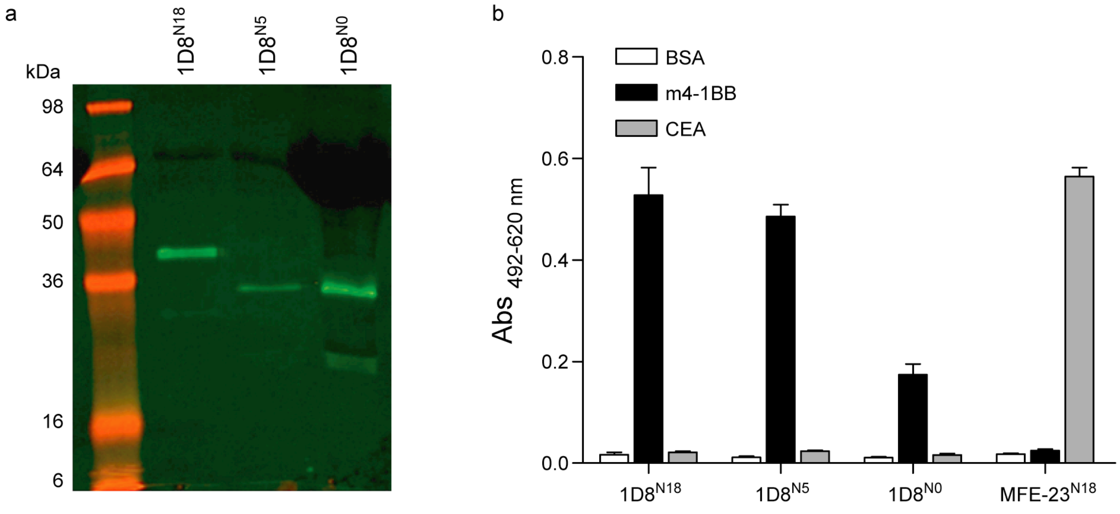

Supplementary Figure 1. Characterization of anti-4-1BB trimerbodies in conditioned media. (**a**) The presence of secreted 1D8^{N18}, 1D8^{N5} and 1D8^{N0} trimerbodies in the conditioned media from transfected HEK293 cells was demonstrated by western blot analysis. Migration of molecular mass markers is indicated (kDa). (**b**) The functionality of secreted 1D8^{N18}, 1D8^{N5}, 1D8^{N0} and MFE-23^{N18} trimerbodies was demonstrated by ELISA against plastic immobilized m4-1BB and human CEA. Data is expressed as a mean ± SD (n = 3) of one representative experiment.

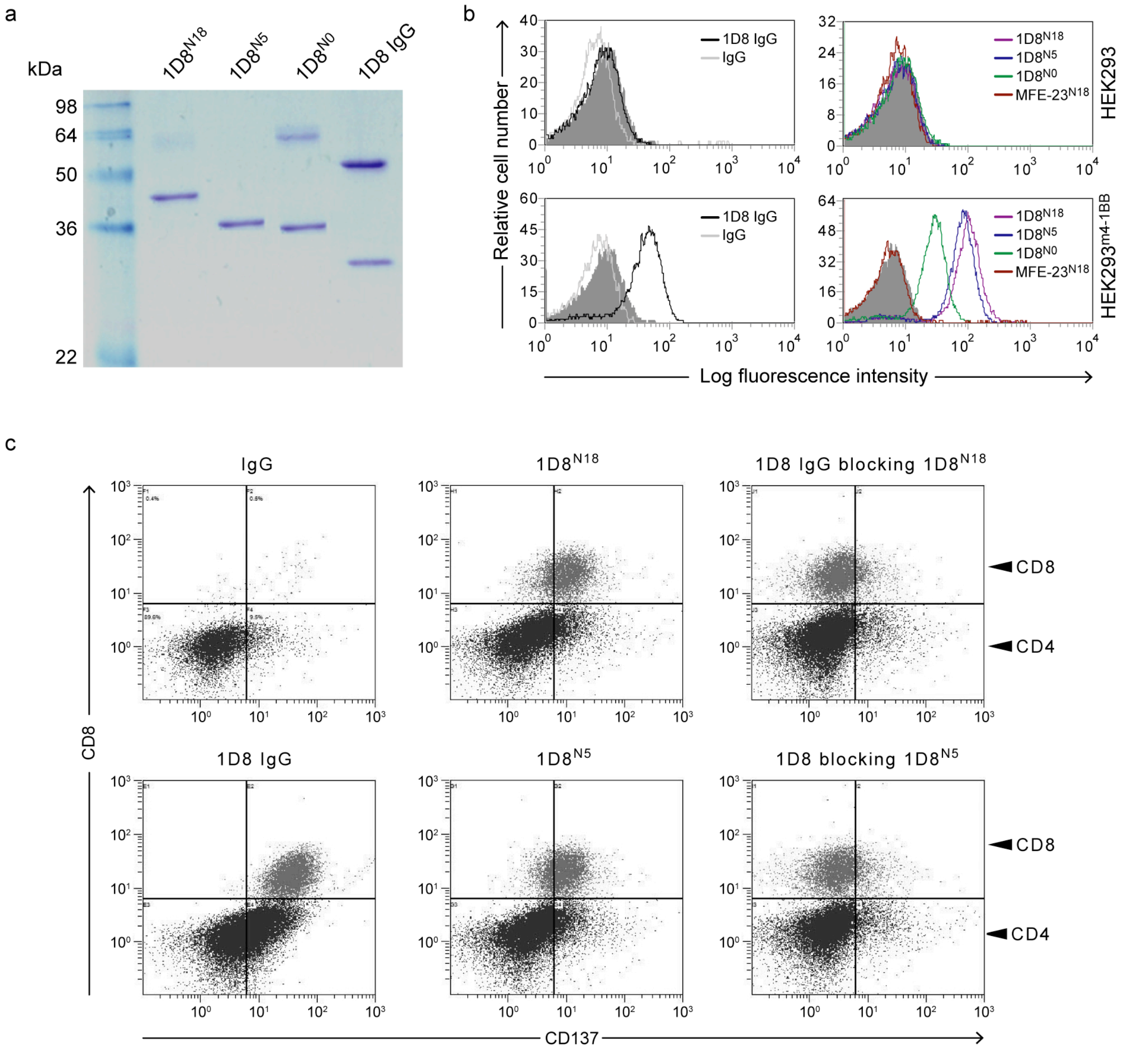

Supplementary Figure 2. Functional characterization of the anti-4-1BB trimerbodies. (**a**) Reducing SDS-PAGE of the purified 1D8^{N18}, 1D8^{N5} and 1D8^{N0} and 1D8 IgG. (**b**) Functional characterization of the 1D8^{N18}, 1D8^{N5} and 1D8^{N0} by FACS on m4-1BB-negative HEK293 cells and on m4-1BB-positive HEK293$^{m4\text{-}1BB}$ cells. The 1D8 IgG and the MFE-23^{N18} trimerbody were used as controls. The y-axis shows the number of cells and the x-axis represents the intensity of fluorescence, expressed on a logarithmic scale. One representative experiment out of three independent experiments is shown. (**c**) 1D8 IgG outcompete binding of 1D8^{N5} and 1D8^{N18} to activated mouse T cells. CD3^{+} T cells were gated and binding of 1D8^{N5}, 1D8^{N18} and 1D8 IgG to CD137 (4-1BB) was determined by FACS. Blockage of 1D8^{N5} and 1D8^{N18} binding by preincubation with 1D8 IgG is shown. T cell staining with anti-CD8 mAb was also performed to discriminate between CD4 and CD8 T cells.

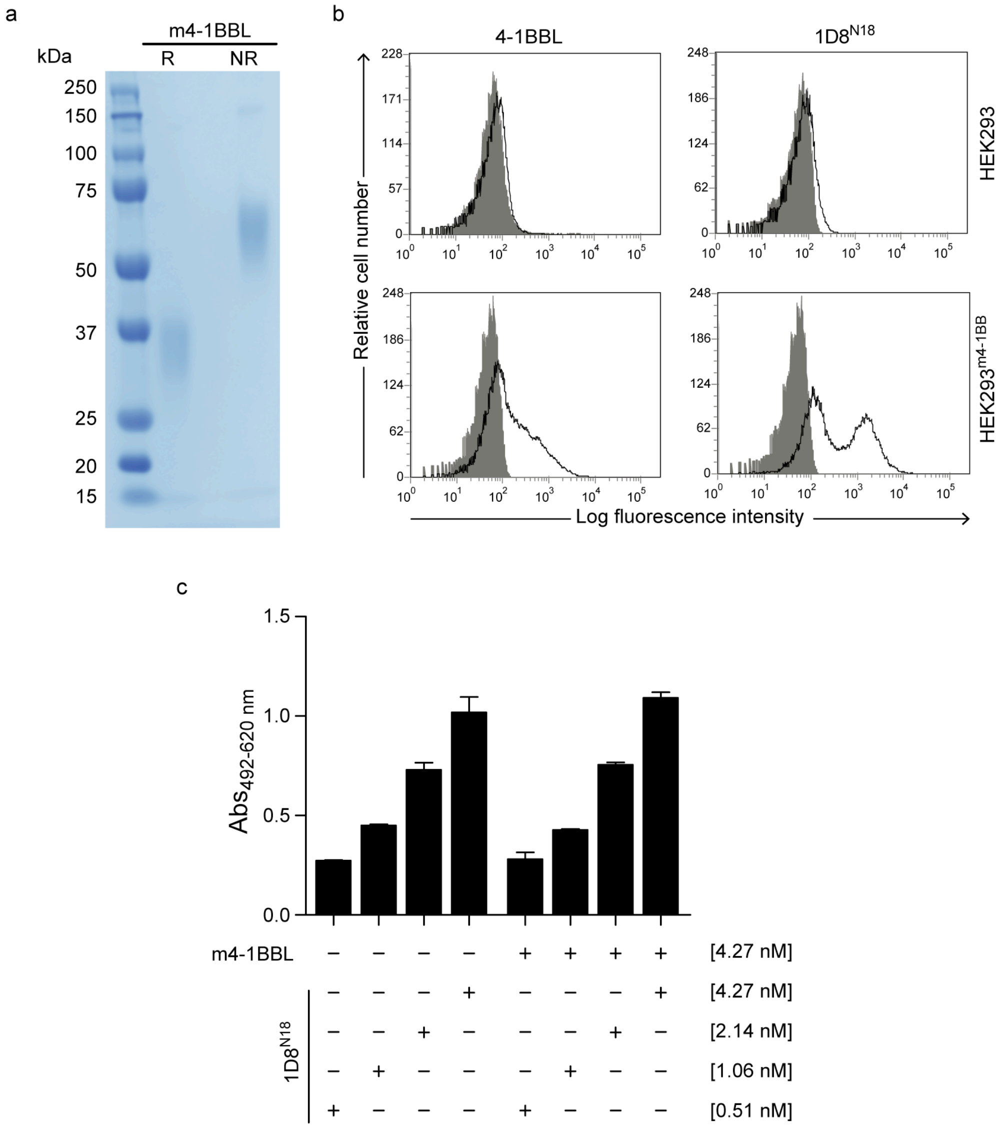

Supplementary Figure 3. Characterization of purified mouse 4-1BBL. (**a**) Coomassie-stained SDS-PAGE of purified recombinant mouse 4-1BBL (m4-1BBL) in both reducing and non-reducing conditions. (**b**) Functional characterization of the m4-1BBL by FACS. The $1D8^{N18}$ trimerbody was used as control. The y-axis shows the number of cells and the x-axis represents the intensity of fluorescence, expressed on a logarithmic scale. One representative experiment out of three independent experiments is shown. (**c**) The functional ability of m4-1BBL to block binding of $1D8^{N18}$ to immobilized m4-1BB was measured in a competitive ELISA with fixed concentration of m4-1BBL and serial dilutions of $1D8^{N18}$. The data shown are expressed as mean ± SD of one representative experiment from a total of three.

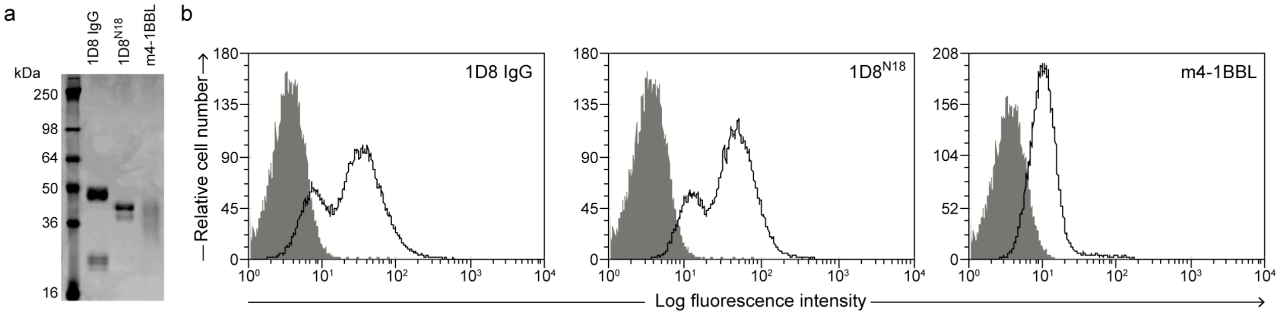

Supplementary Figure 4. Characterization of CF488A-labeled antibodies. (**a**) Coomassie-stained SDS-PAGE of CF488A-labeled 1D8 IgG, $1D8N^{18}$ and m4-1BBL in reducing conditions. (**b**) Functional characterization of CF488A-labeled 1D8 IgG, $1D8N^{18}$ and m4-1BBL by FACS on $HEK293^{m4\text{-}1BB}$ cells. The y-axis shows the number of cells and the x-axis represents the intensity of fluorescence, expressed on a logarithmic scale. One representative experiment out of three independent experiments is shown.

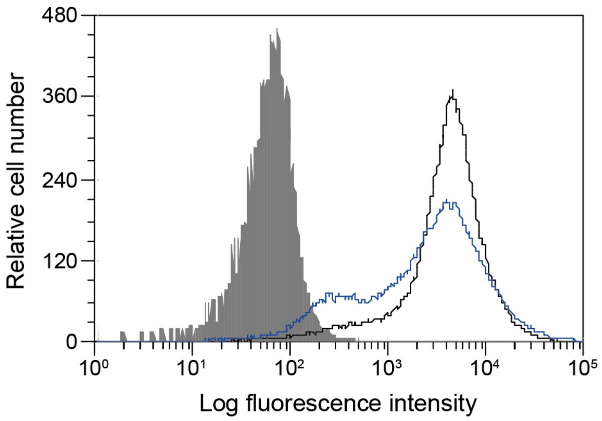

Supplementary Figure 5. FACS analysis of HEK293 cells expressing m4-1BB. Expression profile of original HEK293$^{m4\text{-}1BB}$ cells (pre-sorting) is shown in blue line, and sorted HEK293 cells expressing homogeneous levels of 4-1BB (HEK293$^{m4\text{-}1BB}$-S) is shown in black line. Cells incubated with PE-conjugated isotype control antibodies are shown as grey-filled histogram. The y-axis shows the number of cells and the x-axis represents the intensity of fluorescence, expressed on a logarithmic scale. One representative experiment out of three independent experiments is shown.

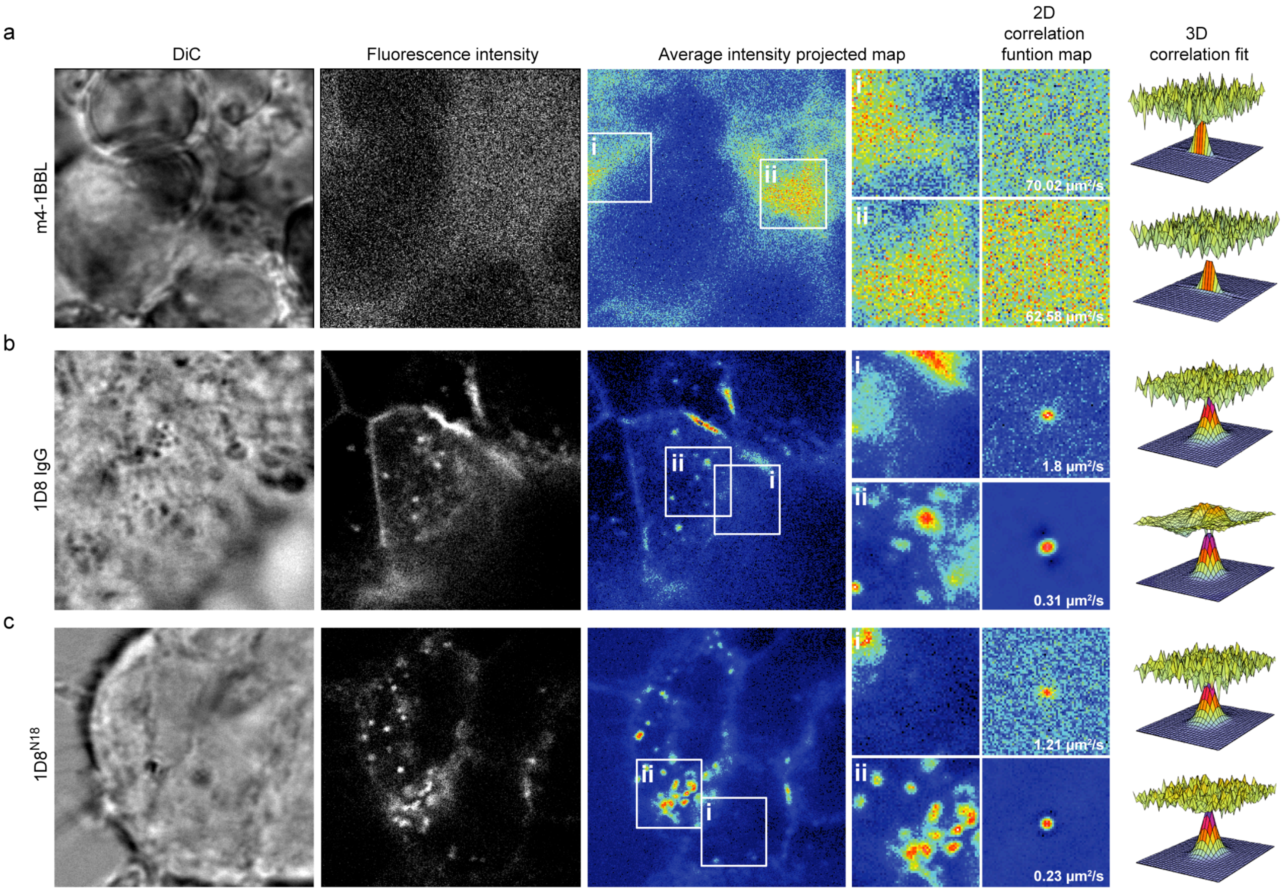

Supplementary Figure 6. Receptor diffusion coefficient in the plasma membrane is much lower upon clustering by 1D8^{N18} trimerbody binding. RICS analyses show the molecular mobility at the plasma membrane of HEK293$^{m4\text{-}1BB}$-S cells, after 100 ng/ml addition of either recombinant m4-1BBL (**a**), 1D8 IgG (**b**), or 1D8^{N18} (**c**). From left to right: differential interference contrast images (DIC); fluorescence intensity images of the plasma membrane region investigated; average intensity projected map of the exact same region, showing 2 white solid squared highlighted regions (i and ii; where i region showed less or non-clustering than region ii); zoomed-out regions of interest i and ii; 2D autocorrelation function map and the respective diffusion coefficient obtained from the fitting; 3D correlation fit surface plot of the resulted fitting. Analyses were performed over 150 frames (~5 min, 2 seconds per frame). For scale purposes, the pixel size is 80.4 nm and the full frame image is 256x256 pixels, while the zoomed in regions are 64x64 pixels.

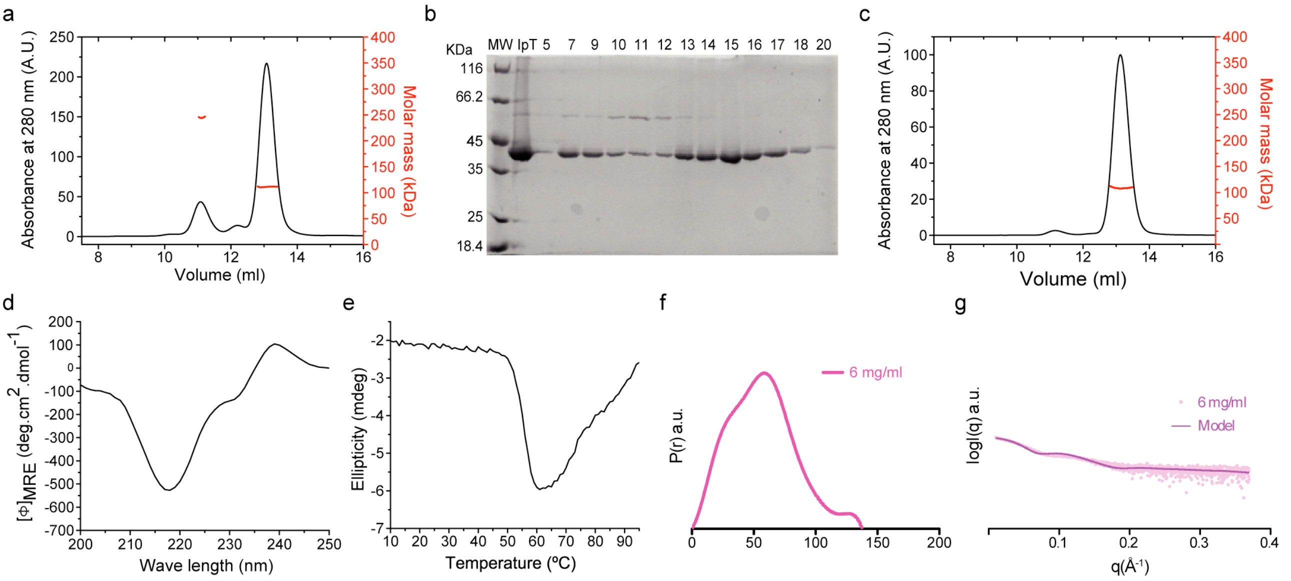

Supplementary Figure 7. Structural characterization of the 1D8^{N18} trimerbody. (**a**) SEC-MALS. The black line corresponds to the UV absorbance (left axis) and the red line to the measured molar mass (right axis). From left to right the peaks correspond to hexamers (16 % of the total intensity), an unknown minor impurity (3 %), and trimers (81%). (**b**) SDS-PAGE analysis of the separation of 1D8^{N18} hexamers and trimers by semipreparative SEC. MW are the molecular weight markers, IpT is the material injected in the column, and the numbers correspond to the chromatogram fractions. (**c**) SEC-MALS analysis of fraction 14 of the 1D8^{N18} SEC chromatogram analyzed in **b**. The black lines correspond to the UV absorbance (left axis) and the red line to the measured molar mass (right axis). The peak corresponding to the trimer accounts for 97% of the total intensity. Circular dichroism spectrum (**d**) and (irreversible) thermal denaturation (**e**) of 1D8^{N18} measured by the change in circular dicroism ellipticity at 218 nm. (**f**) Normalized pair-distance distribution function P(r) for 1D8^{N18} at 6 mg/ml. The data were offset vertically for clarity. a.u. (arbitrary units). (**g**) SAXS experimental scattering data (dots) and theoretical scattering computed from the models (smooth curves).

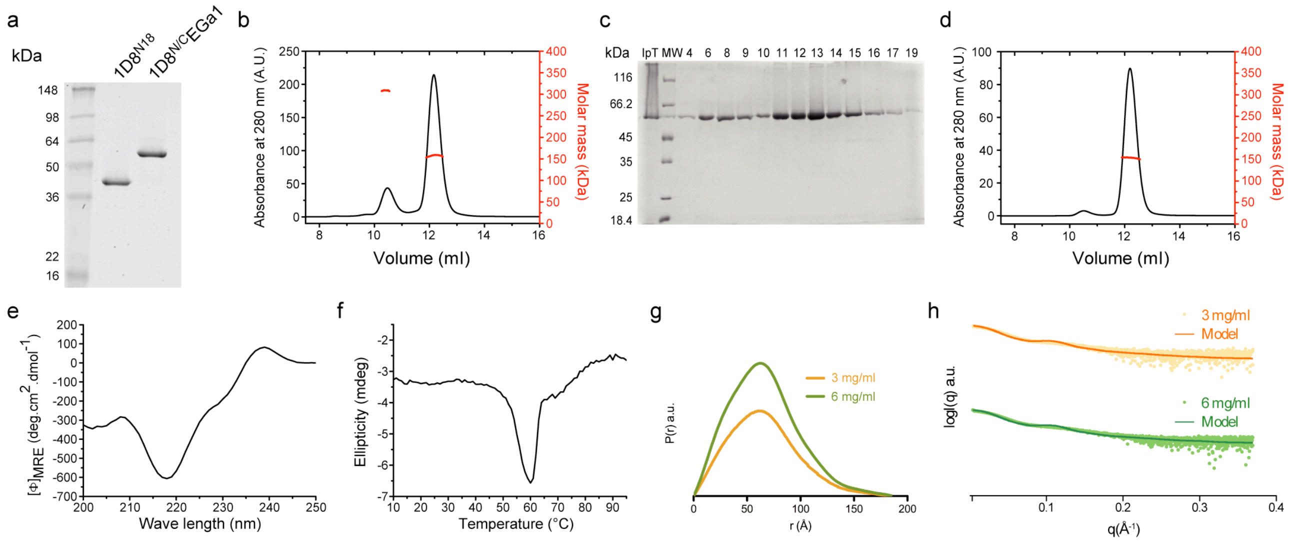

Supplementary Figure 8. Structural characterization of the 1D8$^{N/C}$EGa1 trimerbody. (**a**) Reducing SDS-PAGE of purified 1D8^{N18} and 1D8$^{N/C}$EGa1. (**b**) SEC-MALS analysis 1D8$^{N/C}$EGa1 with the indicated molecular masses measured at the center of the chromatography peaks. The black line corresponds to the UV absorbance (left axis) and the red line to the measured molar mass (right axis). From left to right the peaks correspond to hexamers (16% of the total intensity), and trimers (84%). (**c**) SDS-PAGE analysis of the separation of 1D8$^{N/C}$EGa1 hexamers and trimers by semipreparative SEC. MW are the molecular weight markers, IpT the material injected in the column, and the numbers correspond to the chromatogram fractions. (**d**) SEC-MALS analysis of fraction 12 of the 1D8$^{N/C}$EGa1 SEC chromatogram analyzed in **b**. The black lines correspond to the UV absorbance (left axis) and the red line to the measured molar mass (right axis). The peak corresponding to the trimer accounts for 97% of the total intensity. Circular dichroism spectrum (**e**) and (irreversible) thermal denaturation (**f**) of 1D8$^{N/C}$EGa1 measured by the change in circular dicroism ellipticity at 218 nm. (**g**) Normalized pair-distance distribution function P(r) for 1D8$^{N/C}$EGa1 at 3 and 6 mg/ml (orange and green graphs). The data were offset vertically for clarity. a.u. (arbitrary units). (**h**) SAXS experimental scattering data (dots) and theoretical scattering computed from the models (smooth curves) at the two concentrations.

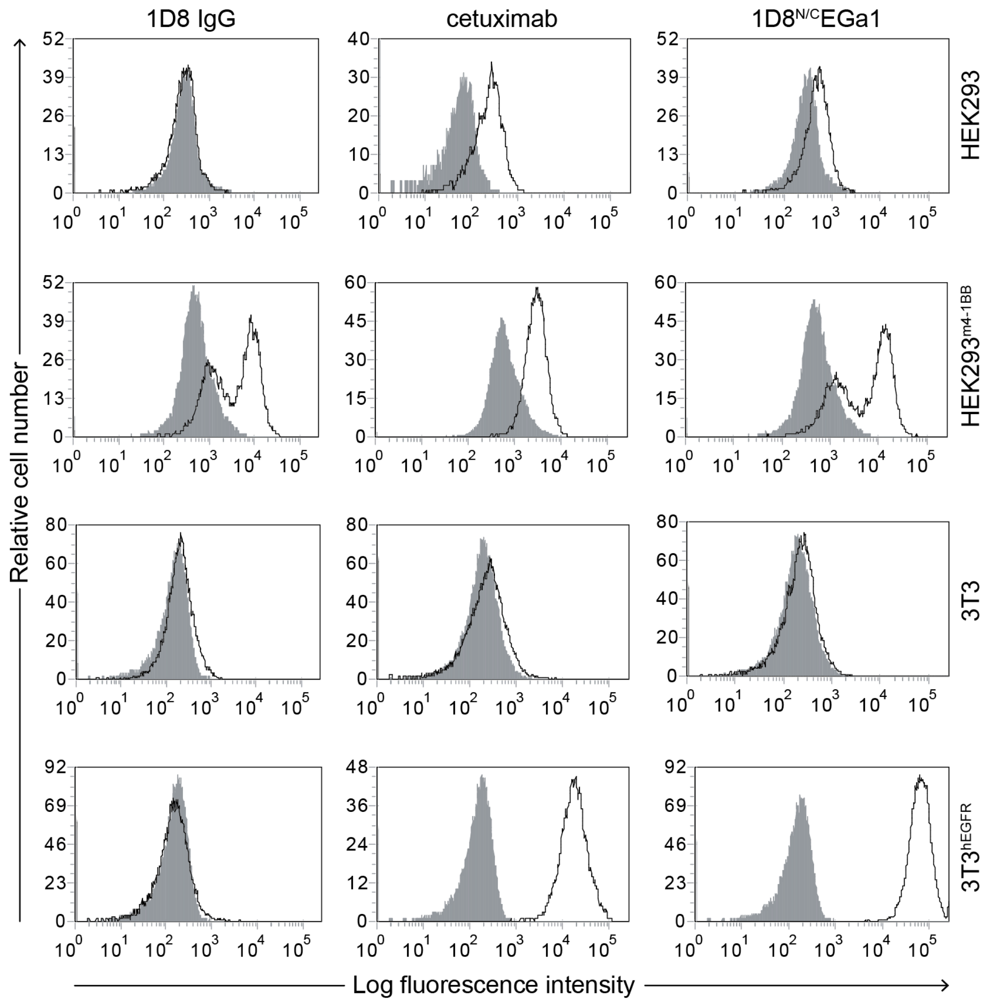

Supplementary Figure 9. Functional characterization of the 1D8$^{N/C}$EGa1 trimerbody by FACS. The 1D8 IgG and cetuximab were used as controls. The y-axis shows the relative number of cells and the x-axis represents the intensity of fluorescence, expressed on a logarithmic scale. One representative experiment out of three independent experiments is shown.

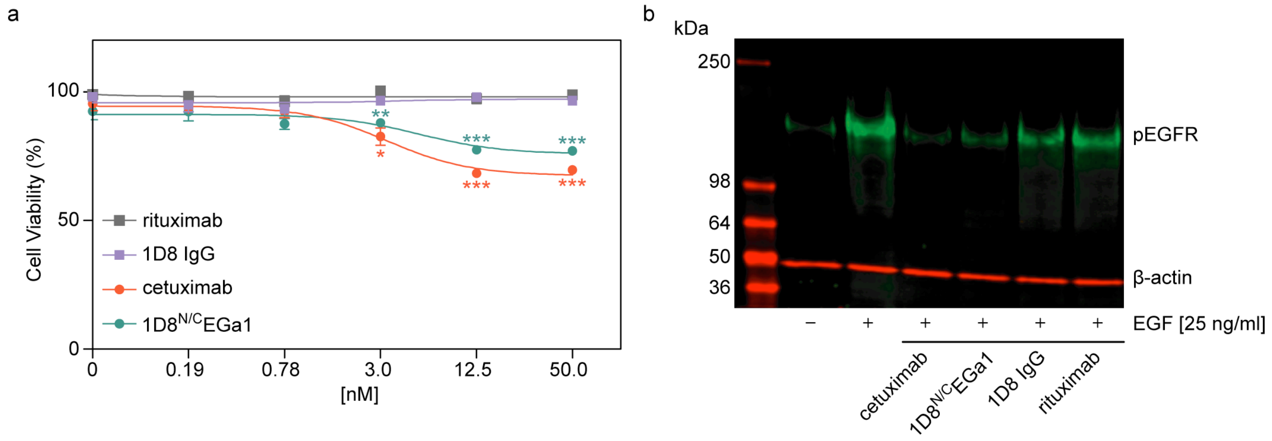

Supplementary Figure 10. Effect of 1D8$^{N/C}$EGa1 trimerbody on EGFR-mediated signaling. (**a**) Inhibition of A431 cell proliferation**.** The cells were treated with the indicated doses of 1D8$^{N/C}$EGa1, 1D8 IgG, cetuximab (positive control) or rituximab (negative control). Viable cells were measured in triplicates after 72 hours of treatment and plotted relative to untreated controls. Results are expressed as a mean ± SD ($n = 3$). Significance was measured by unpaired Student's t test; *$P \leq 0.05$, **$P \leq 0.01$, ***$P \leq 0.001$ (green asterisks, comparison of 1D8$^{N/C}$EGa1 with rituximab; red asterisks, comparison of cetuximab with rituximab). (**b**) Inhibition of EGFR phosphorylation. Cells were pre-incubated with 50 nM of each antibody 4 hours prior to stimulation for 5 minutes with EGF or vehicle. Phosphorylation status of EGFR was assessed by Western Blotting.

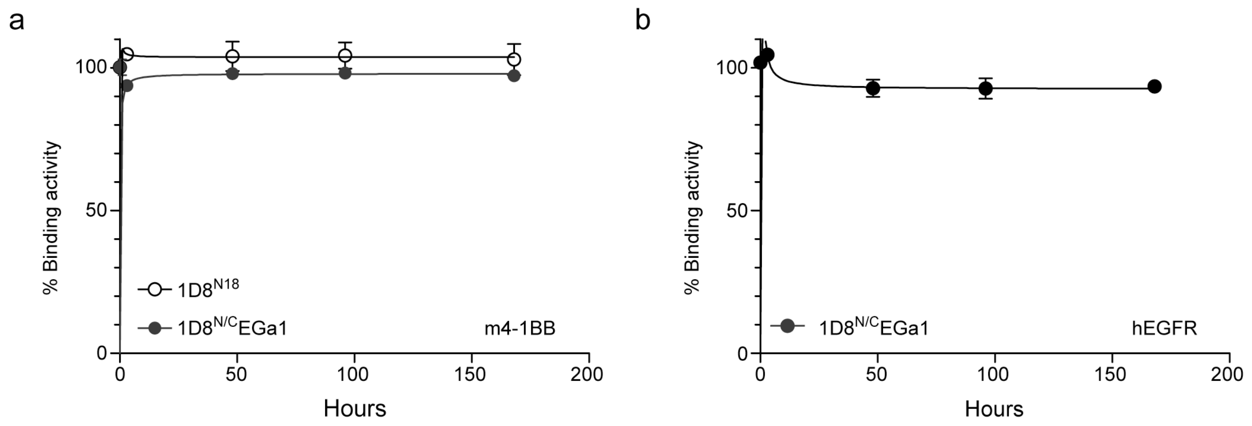

Supplementary Figure 11. Serum stability of purified 1D8^{N18} and 1D8$^{N/C}$EGa1 trimerbodies. ELISA against plastic immobilized m4-1BB (**a**) or hEGFR (**b**) was performed after incubation at 37 °C for different time periods in human serum. Mean ± SD are shown at each time point. *P* values were determined by unpaired Student's *t* test (*$P \leq 0.05$, **$P \leq 0.01$, ***$P \leq 0.001$).

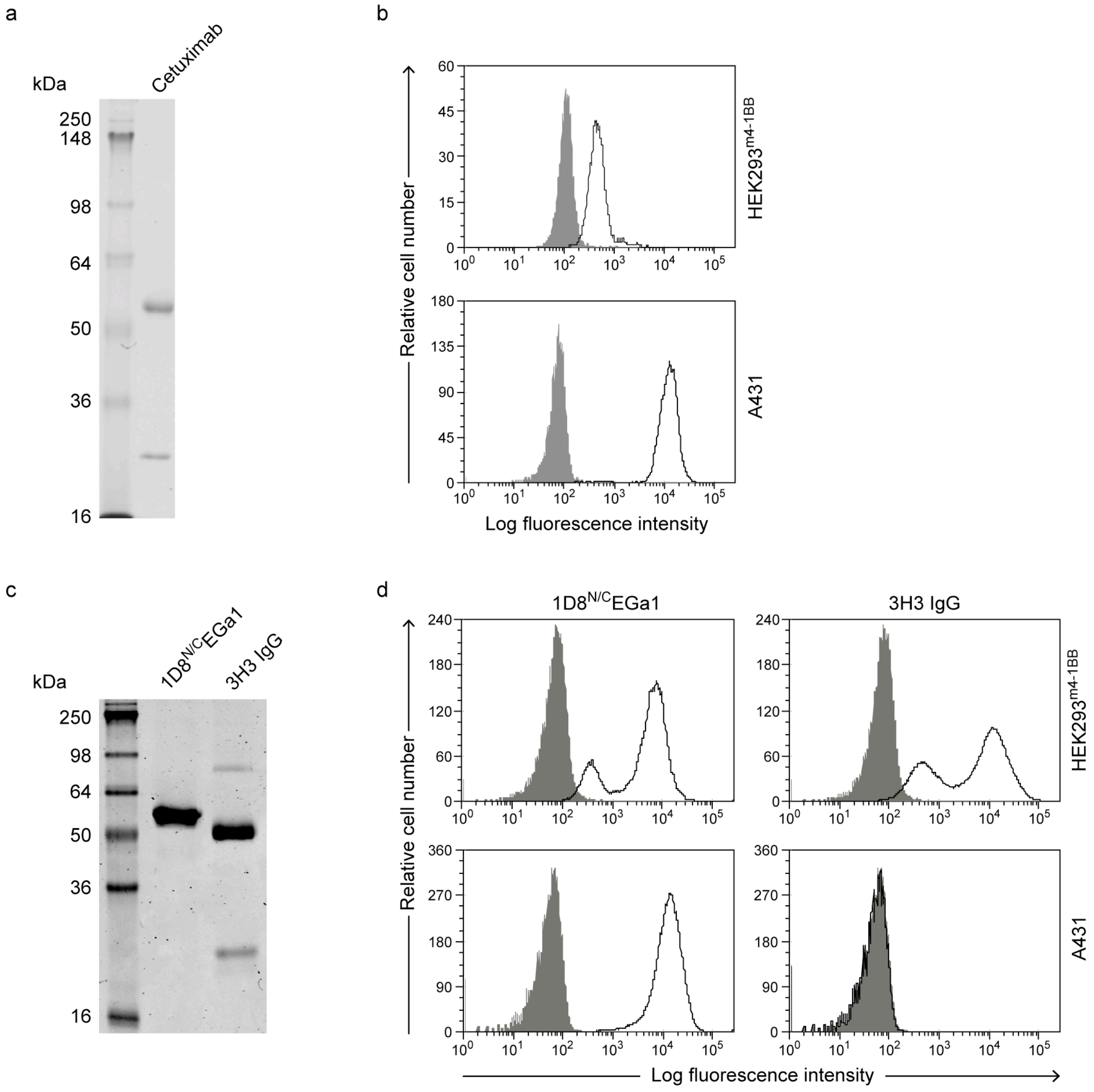

Supplementary Figure 12. Characterization of antibodies labeled with near-infrared fluorochromes. (**a**) Coomassie stained SDS-PAGE gel in reducing conditions of Cy5-labeled cetuximab. (**b**) Functional characterization of Cy5-labeled cetuximab by FACS on HEK293^{m4-1BB} and A341 cells. The y-axis shows the number of cells and the x-axis represents the intensity of fluorescence, expressed on a logarithmic scale. (**c**) Coomassie stained SDS-PAGE gel in reducing conditions of CF647-labeled 1D8$^{N/C}$EGa1 and 3H3 IgG. (**d**) Functional characterization of CF647-labeled 1D8$^{N/C}$EGa1 and 3H3 IgG by FACS on HEK293^{m4-1BB} and A341 cells. One representative experiment out of three independent experiments is shown.

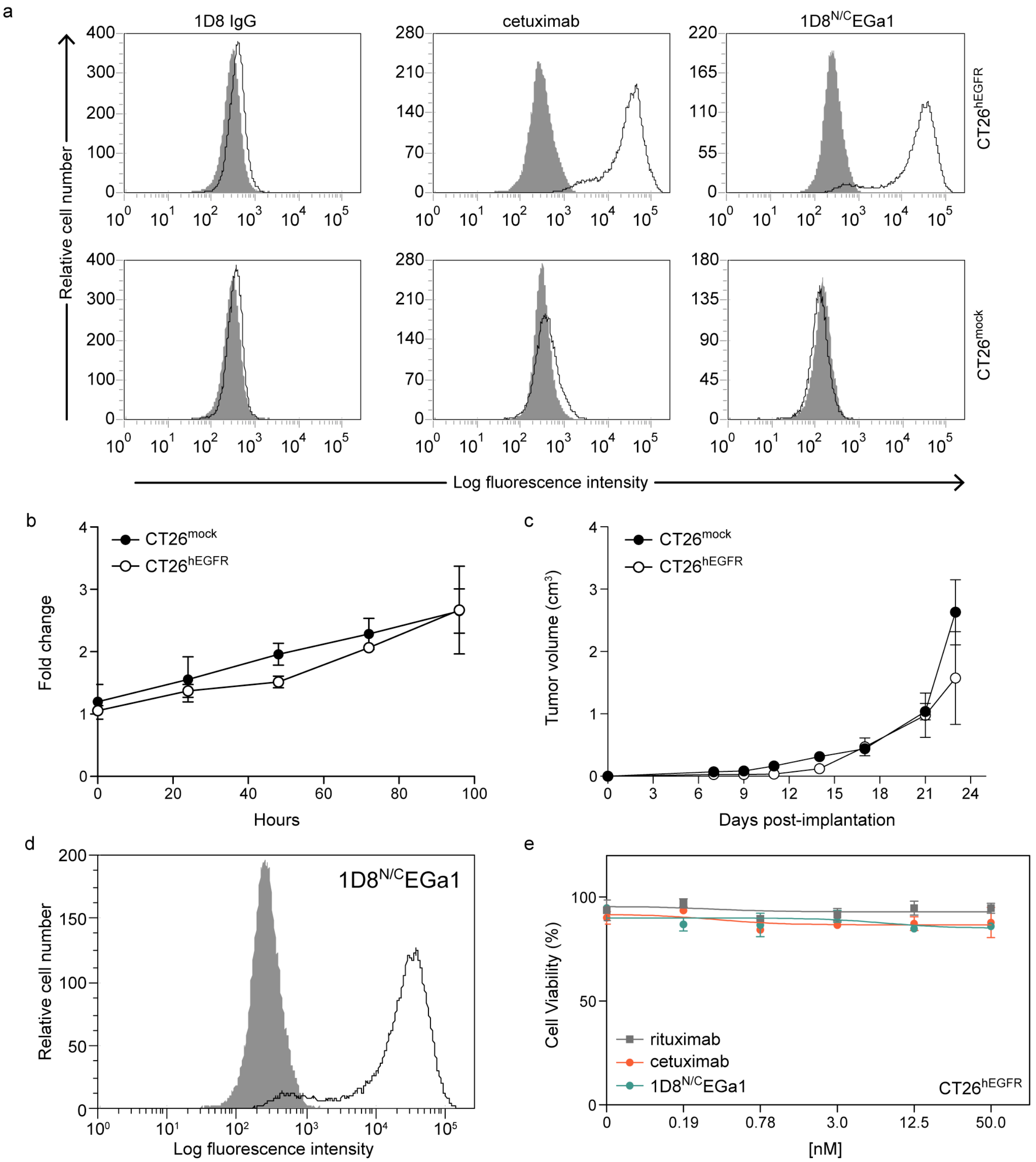

Supplementary Figure 13. Characterization of CT26^{hEGFR} and CT26^{mock} cells. (**a**) FACS analysis of 1D8$^{N/C}$EGa1 trimerbody binding to the surface of CT26 EGFR-expressing cells (CT26^{hEGFR}) and CT26 infected with the empty vector retrovirus (CT26^{mock}). The 1D8 IgG and cetuximab were used as controls. The y-axis shows the number of cells and the x-axis represents the intensity of fluorescence, expressed on a logarithmic scale. One representative experiment out of three independent experiments is shown. (**b**) Comparative analysis of *in vitro* cell proliferation of CT26^{hEGFR} versus CT26^{mock} cells by the Cell Titer-Glo assay. Data represent the mean ± SD of three

independent experiments, and are presented as fold change relative to initial value. (**c**) Comparative *in vivo* tumor growth of $CT26^{mock}$ versus $CT26^{hEGFR}$ monitored up to 30 days. Tumor volume (cm^3) is expressed as a mean ± SD (n = 5) of one representative experiment and P values were determined by unpaired Student's t test. (**d**) $CT26^{hEGFR}$ tumors were extracted after 30 days, mechanically dissociated and single cell suspensions analyzed for EGFR expression by FACS. Results from one representative animal are shown (n = 5). (**e**) The $1D8^{N/C}EGa1$ trimerbody is not an inhibitor of $CT26^{hEGFR}$ proliferation. $CT26^{hEGFR}$ cells were incubated in the presence of equimolar concentrations of $1D8^{N/C}EGa1$, cetuximab and rituximab, and their effect on proliferation is represented as percentage compared to controls. Data represent mean ± SD (n = 5) and P values were determined by unpaired Student's t test.

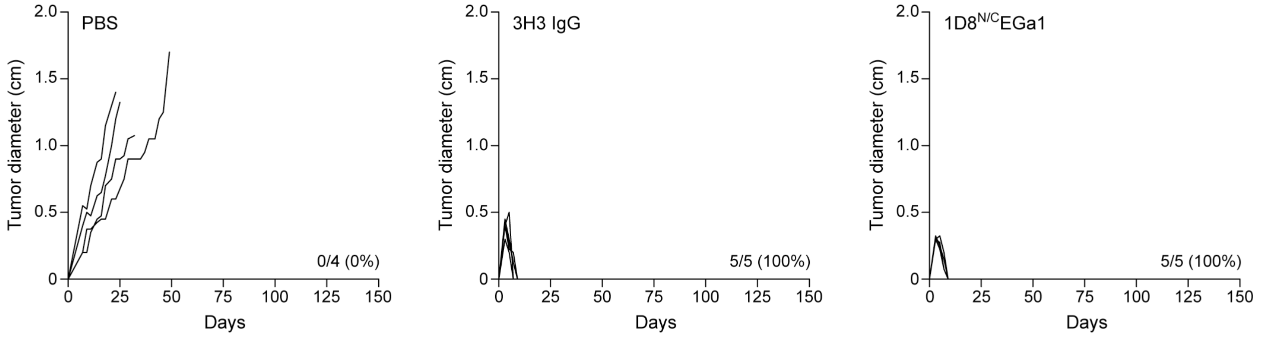

Supplementary Figure 14. Long-lasting systemic immune-mediated response after systemic injections of 3H3 IgG or 1D8$^{N/C}$EGa1 trimerbody. Long-termed survivors (*n* = 5 mice/group), following complete tumor rejection were re-challenged with 1.5x10^{6} CT26^{mock} cells (s.c.) 50 days after i.p. injections of 1D8$^{N/C}$EGa1 trimerbody or 3H3 IgG. As a control group, tumor naïve mice developed tumors in every case. Tumor diameter growth curves for individual mice in each treatment group are presented.

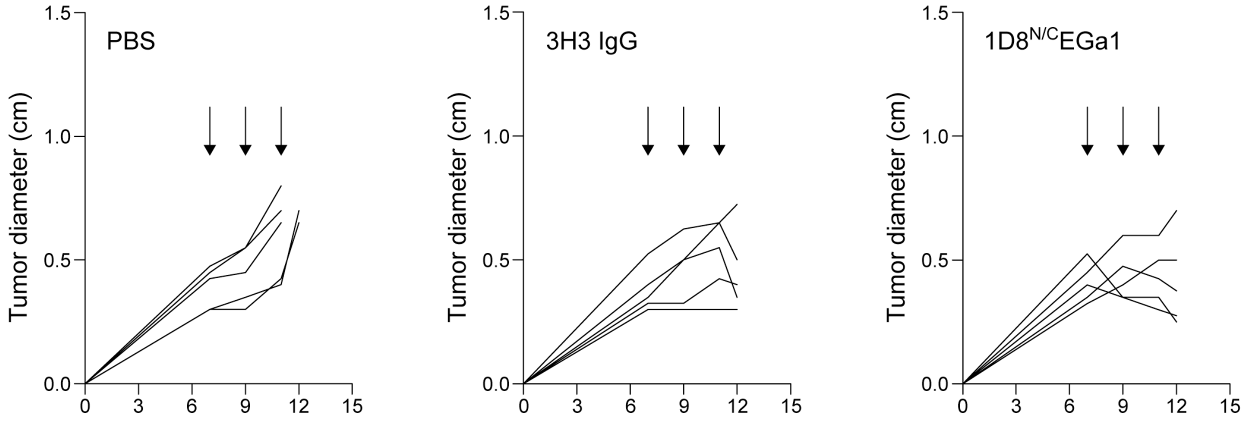

Supplementary Figure 15. Induction of tumor regression in mice treated with 3H3 IgG and 1D8$^{N/C}$EGa1 trimerbody. BALB/c mice (*n* = 5/group) were inoculated s.c. with CT26^{hEGFR} tumor cells and treated (black arrows) every other day with three i.p. injections (4 mg/kg) of 3H3 IgG, 1D8$^{N/C}$EGa1, or with PBS and monitored for tumor growth. Tumor diameter growth curves for individual mice in each treatment group are presented. At day 13 mice were euthanized and tumors collected, formalin-fixed, paraffin-embedded and stained by immunohistochemistry.

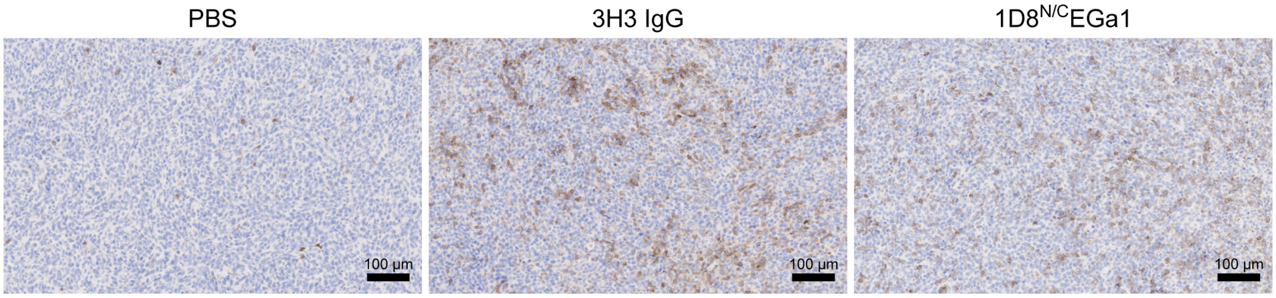

Supplementary Figure 16. Representative examples of CD8+ TILs immunostaining, in mice treated with PBS, 3H3 IgG, and 1D8$^{N/C}$EGa1. Scale bars are shown.

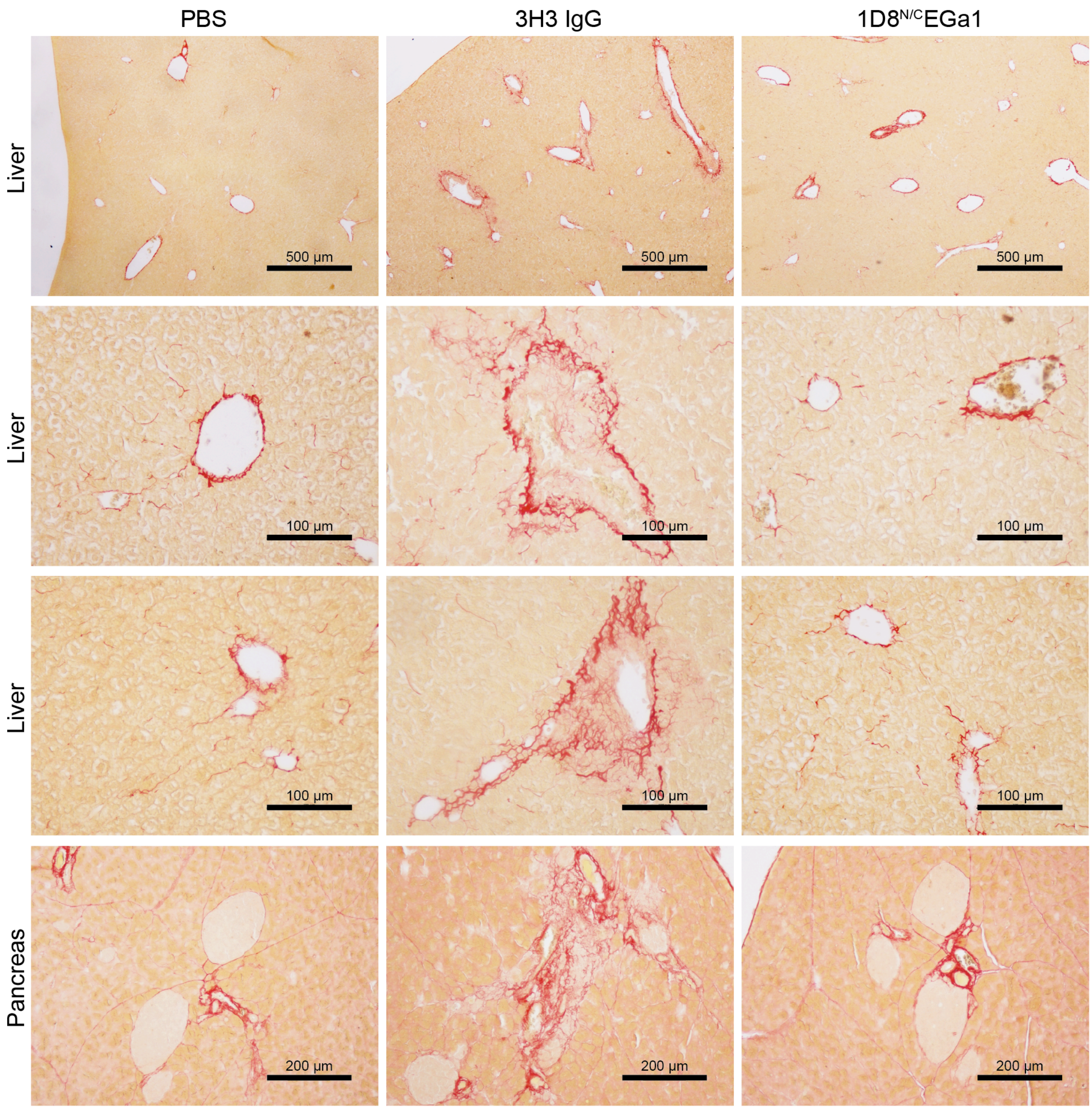

Supplementary Figure 17. Sirius red staining of collagen fibers of representative tissue slides from liver and pancreas of mice treated with PBS, 3H3 IgG or 1D8$^{N/C}$EGa1. Magnification is 40X and 200X (liver) and 100X (pancreas). Scale bars are shown.

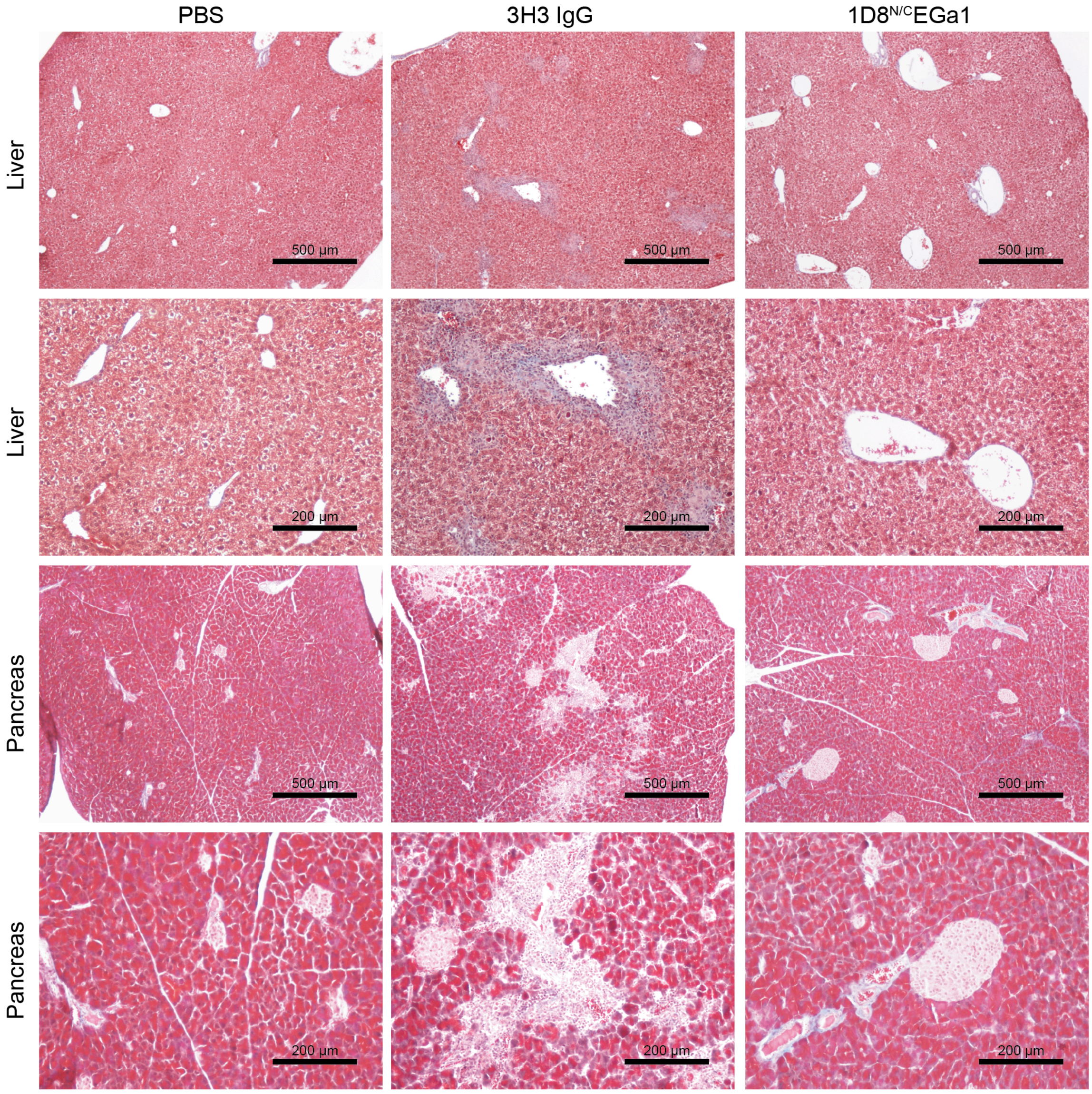

Supplementary Figure 18. **Masson trichrome staining of representative tissue slides** from liver and pancreas of mice treated with PBS, 3H3 IgG or 1D8$^{N/C}$EGa1 Magnification is 40X and 100X. Scale bars are shown.

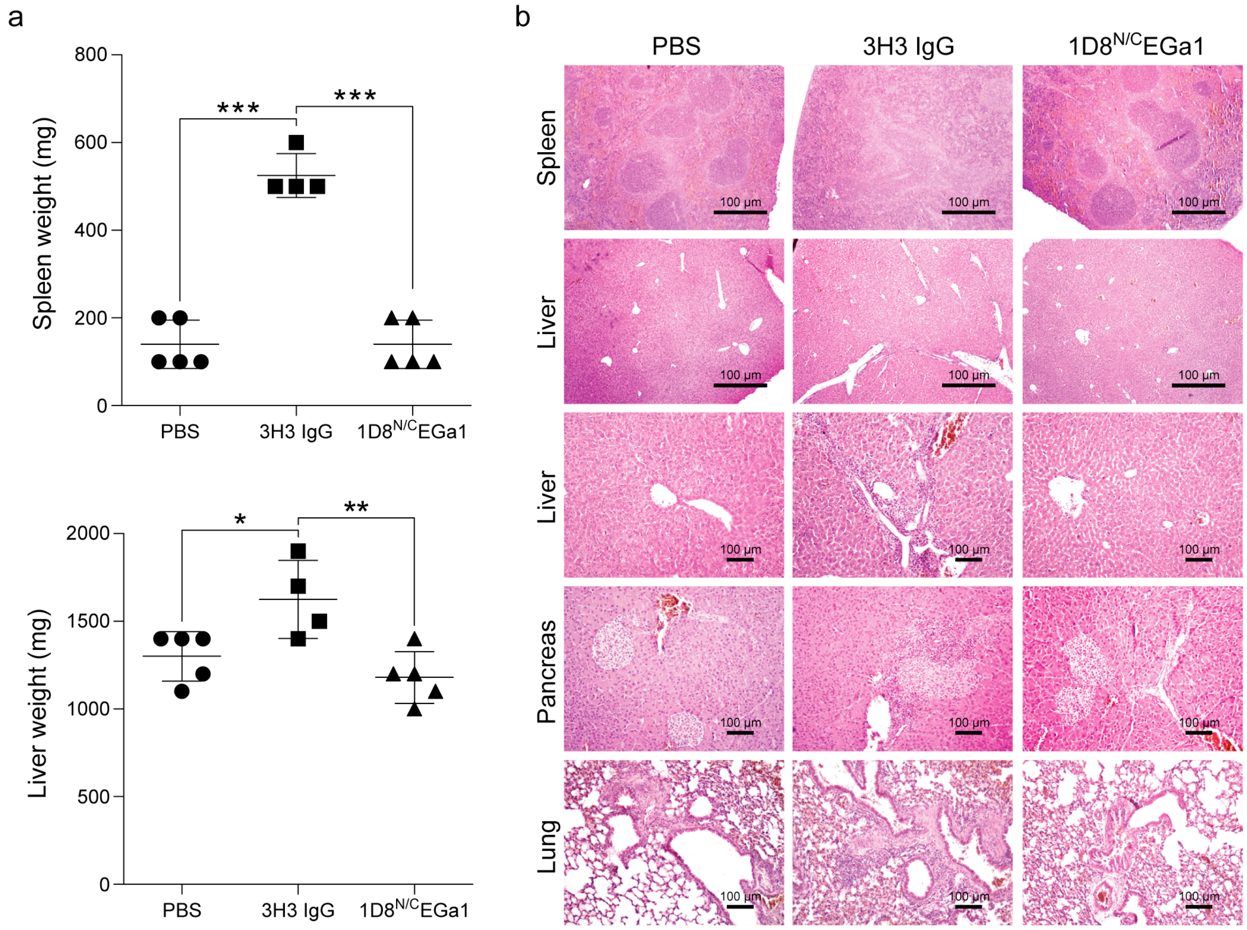

Supplementary Figure 19. Treatment with 1D8$^{N/C}$EGa1 every 3 days does not induce toxicity. (**a**) Spleen and livers weights from mice (n = 5/group), treated with PBS, 3H3 IgG, or 1D8$^{N/C}$EGa1 are shown. Data are mean ± SD, *$P \le 0.05$, **$P \le 0.01$, ***$P \le 0.001$, Student t-test. (**b**) Hematoxylin and eosin staining of representative tissue slides from spleen, liver, pancreas, and lung of mice treated with PBS, 3H3 IgG, and 1D8$^{N/C}$EGa1. Magnification is 40 X (spleen and liver) and 200 X (liver, pancreas and lung). Scale bars are shown.

Supplementary Table 1. Kinetic rate constants and K_D values derived from fitting BLI data to a 1:1 binding model.

Antigen	Antibody	$K_D(pM)$	$k_a(M^{-1}s^{-1})$	$k_d(s^{-1})$
m4-1BB	1D8 IgG	99	$2.2 * 10^5$	$22.2 * 10^{-6}$
	$1D8^{N18}$	60	$1.5 * 10^5$	$9.2 * 10^{-6}$
	$1D8^{N5}$	56	$1.5 * 10^5$	$8.5 * 10^{-6}$
	$1D8^{N0}$	86	$1.3 * 10^5$	$10.8 * 10^{-6}$
	$1D8^{N/C}$EGa1	25	$3.1 * 10^5$	$7.7 * 10^{-6}$
hEGFR	$1D8^{N/C}$EGa1	30	$13.6 * 10^5$	$40 * 10^{-6}$
	$EGa1^{N18}$	167	$5.9 * 10^5$	$9.8 * 10^{-6}$

Supplementary Table 2. SAXS Data collection and derived parameters

**Data collection parameters**			
Instrument	Diamond Light Source beamline B21 (Harwell Campus, UK)		
Wavelength (Å)	1		
q-range ($Å^{-1}$)	0.02–0.45		
Exposure time (s)	300		
Concentration range (mg ml^{-1})	3.6		
Temperature (K)	277		
**Structural parameters**			
Protein / Concentration (mg ml^{-1})	$1D8^{N18}$	$1D8^{N/C}$EGa1 1/3	$1D8^{N/C}$EGa1 1/6
Rg (Å) (from Guinier)	42±0.1	51±0.1	51±0.1
Rg (Å) (from P(r))	44±0.2	51±0.1	52±0.4
Dmax (Å)	153±0.2	183±0.4	185±0.2
Molecular mass determination			
MM (kDa) from Porod volume	146	211	209
Calculated MM (kDa) from	118	159	159
**Software employed**			
Data processing	Scåtter/PRIMUS / GNOM		
Ab initio analysis / Averaging	DAMMIF, DAMMIN / DAMAVER		
Computation of model intensities	FoXS		
Computation of molecular weight	SAXSMoW		
3D graphics representations	PyMOL		

Supplementary Table 3. Pharmacokinetic properties of $1D8^{N18}$ and $1D8^{N/C}EGa1$ trimerbodies after single i.v. dosing of CD-1 mice.

Trimerbody	MW[kDa]	$t_{1/2}\alpha$(hours)	$t_{1/2}\beta$(hours)	AUC µg*h/ml
$1D8^{N18}$	118.2	0.24±2.9	1.27±0.54	2.48
$1D8^{N/C}EGa1$	158.7	1.09±0.64	16.15±0.04	58.33

Supplementary Table 4. Pharmacokinetic properties of 3H3 IgG and $1D8^{N/C}EGa1$ trimerbody after single i.v. dosing of BALB/c mice.

Antibody	MW[kDa]	$t_{1/2}\alpha$(hours)	$t_{1/2}\beta$(hours)	AUC µg*h/ml
3H3 IgG	150	1.19±0.57	116.15±0.01	198.53
$1D8^{N/C}EGa1$	158.7	0.68±1.01	15.81±0.04	78.13

Supplementary Table 5. Pharmacokinetic properties of 3H3 IgG and $1D8^{N/C}EGa1$ trimerbody after single i.p. dosing of BALB/c mice.

Antibody	MW[kDa]	$t_{1/2}\alpha$(hours)	$t_{1/2}\beta$(hours)	AUC µg*h/ml
3H3 IgG	150	8.29±0.08	169.12±0.01	475.55
$1D8^{N/C}EGa1$	158.7	0.18±3.74	32.04±0.02	162.07

Supplementary Table 6. Oligonucleotides used in this study

Name	Sequence (5´-3´)
LEGA-1	AATTCAGGCGCCGGTGGATCTGGTGGCTCCTCTGGCTCAGA CGGAGCGTCGGGTTCGCGA
Stop-*Xba*I-Rev	GCCGGCTCTAGATTATTATGAGGAGACGGTGAC
FwCMV	CGCAAATGGGCGGTAGGCGTG
RvBGH	TAGAAGGCACAGTCGAGG

Oligonucleotides were synthesized by Thermo Scientific.
